# A High Likelihood of a Disastrous Outcome: A Case Report of a Complication From the Cosmetic Use of Hyaluronic Acid

**DOI:** 10.7759/cureus.70636

**Published:** 2024-10-01

**Authors:** Rawan Almutairi, Sarah Mubarak, Wael Aldaraji

**Affiliations:** 1 Dermatology, Farwaniya Hospital, Ministry of Health, Farwaniya, KWT; 2 Dermatology, Laser and Skin Clinic, Baghdad, IRQ; 3 Dermatology, The Ghanem Clinic, London, GBR

**Keywords:** aesthetic medecine, cosmetic injections, dermatology cosmetology, filler injection, hyaluronic acid filler

## Abstract

Hyaluronic acid (HA) injections are a generally safe procedure, primarily performed to achieve a more aesthetically appealing appearance. Despite the safety of HA filler procedures, complications may arise in some individuals, such as tenderness, lumpiness, swelling, bruising, neurological impairments, embolism, soft-tissue necrosis, irreversible scarring, and baldness. We present a case of a 36-year-old previously healthy female who was injected with HA filler (Juvederm Voluma®) into the nasolabial folds for cosmetic purposes. A few hours later, she reported feeling pain in her right nasolabial region; 27 hours after the injection, the patient presented with a violaceous rash and was diagnosed clinically with arterial compromise due to HA injection 27 hours prior. She was managed with aspirin and hyaluronidase injections into the affected area. The complication resolved after two weeks of follow-up. HA used in our patient was very heavy in its molecular characteristics, and it should not be injected in the nasolabial fold. HA injections can lead to various complications, and both practitioners and patients should be aware of these potential adverse effects. Prompt intervention is required to ensure recovery from vascular occlusion in these patients.

## Introduction

Hyaluronic acid (HA) injections are a generally safe procedure, and these are mainly performed to gain a more aesthetically pleasing look [1]. Larl Meyer gave HA its initial name after discovering this polysaccharide in the "vitreous humour" of the bovine eye. Subsequently, this polysaccharide has been discovered in all species, including humans. It is a naturally occurring glycosaminoglycan that builds the extracellular matrix of the connective tissue [2].

Despite the safety of HA filler procedures, certain complications can occur in some individuals. This can be attributed to the rising popularity of filler injections and the concurrent increase in the number of practitioners with limited experience performing them. The consequences associated with HA encompass a broad spectrum, including various manifestations such as tenderness, lumpiness, swelling, bruising, neurological impairments, embolism, vision impairment, soft-tissue necrosis, irreversible scarring, and baldness [1,2]. The most severe side effects are probably caused by the hydrophilic activity of the large HA filler, which causes arterial blockage and direct damage to the vasculature, thereby stopping the blood flow to the affected area. We present a case of vascular occlusion secondary to the use of HA with a heavier molecular characteristic injected into the nasolabial fold.

## Case presentation

**Figure 1:**
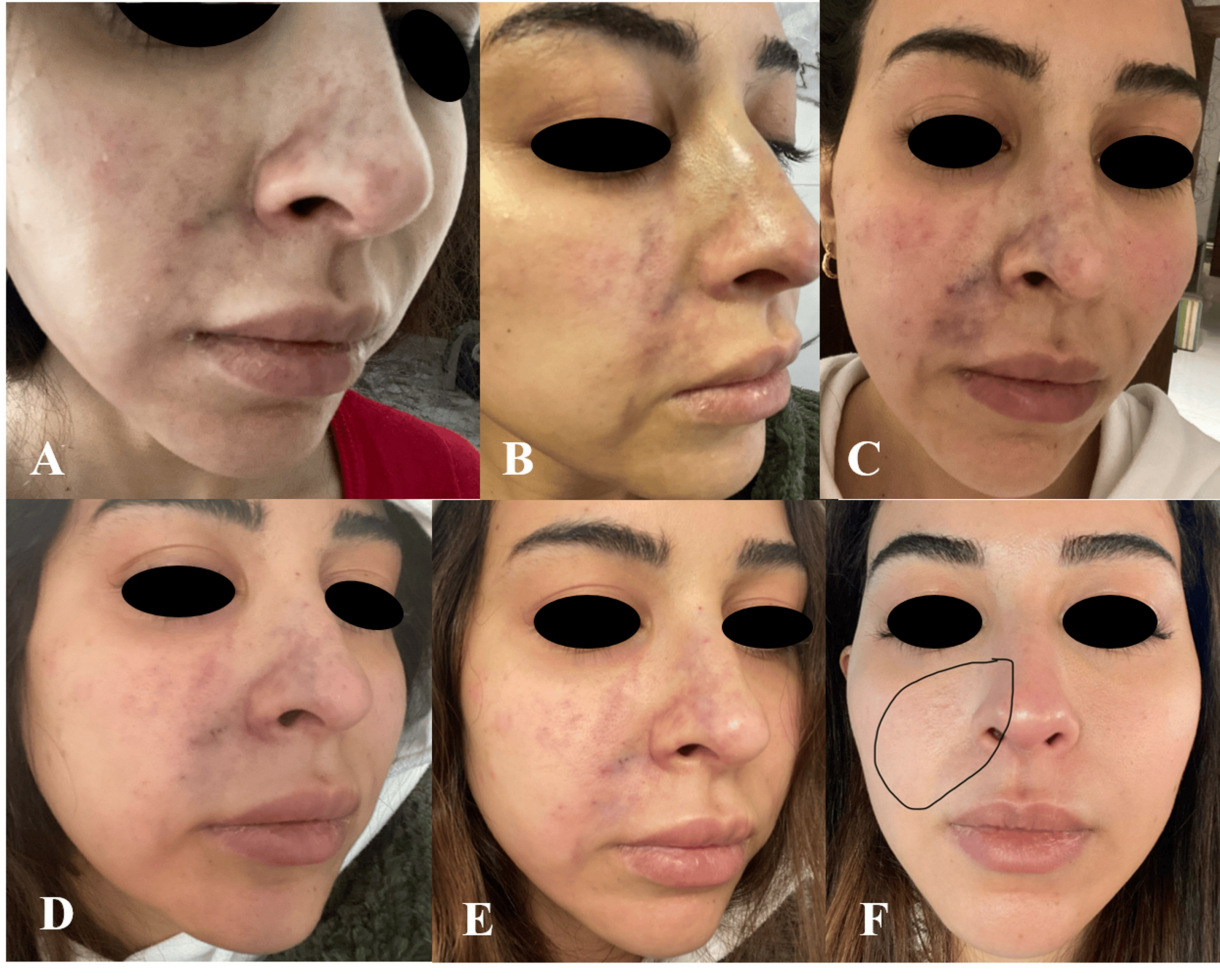
Presentation and stages of the resolution of the violaceous rash due to HA injection into the right nasolabial fold (A) Presentation of a violaceous rash 27 hours after the HA injection. (B, C, D, E, and F) Stages of the rash after administering hyaluronidase injection for three days. (F) Two weeks later, the skin rash completely healed, leaving a few papules that faded away after two more weeks HA: hyaluronic acid

A 36-year-old previously healthy female presented to the dermatology clinic and was injected with HA filler (Juvederm Voluma®) into the nasolabial folds for cosmetic purposes. A few hours later, she reported feeling pain in her right nasolabial region, which was partially relieved with non-steroidal anti-inflammatory medication. Twenty-seven hours after the injection, the patient presented with a violaceous rash (Figure 1) and was diagnosed clinically with arterial compromise due to HA injection. She was managed with aspirin and hyaluronidase injections of 350 units into the affected area every four hours for three days. Two weeks later, the skin rash completely healed, leaving a few papules that faded away after two more weeks.

## Discussion

The nasal region has been designated a high-risk area when injecting HA fillers due to its susceptibility to tissue necrosis, which is attributed to the fact that the nasal region is a specific area that relies exclusively on a single artery branch. Although the occurrence of unintentional intra-arterial injection of dermal fillers is infrequent, it is important to consider the potential complication of vascular embolization while injecting the subcutis of the nasolabial folds [3]. Hence, nasal necrosis could potentially be identified through compression of the facial artery caused by the injection of nasolabial folds or compression of the angular artery at the alar margin [2].

HA injected into a blood vessel can create a physical blockage. The high molecular weight of HA contributes to the formation of a bolus that can obstruct blood flow. This blockage can trigger a prothrombotic state, leading to platelet aggregation and thrombus formation. While the initial bolus may not fully occlude the vessel, it can promote subsequent aggregation of platelets, leading to a larger thrombus that further compromises the blood flow [4].

If compromised blood flow is suspected, the practitioner should promptly cease injecting any further substance. Conservative interventions, such as the application of massage, tapping, or heat to the affected region, may potentially lead to the resolution of the occlusion. Nevertheless, hyaluronidase remains the cornerstone of management. Ischemia is treated by administering hyaluronidase via diffuse injection into the afflicted tissue. Direct injection into the blood vessel is not always necessary if it is obstructed, as hyaluronidase tends to disperse extensively. However, the use of ultrasound-guided hyaluronidase injection may be favored because it allows for precise localization of the filler material.

Aspirin is additionally added as an antiplatelet medication to inhibit the formation of additional blood clots. In cases of necrosis, the use of antibiotics is recommended because of the susceptibility of deceased cells and tissues to subsequent opportunistic infections. Analgesia may be necessary in instances of necrosis due to its potential to induce intense pain. Additional therapy alternatives include the administration of low-molecular-weight heparin to avert thrombosis and embolization, or oral vasodilators to manage vascular blockage. Nevertheless, there is scarce data in the literature to support the widespread use of either medication. Prompt and regular follow-up of these patients is important in all situations with complications [1,5,6].

## Conclusions

HA injections, commonly used in cosmetic procedures, can lead to various complications. Understanding these potential adverse effects is essential for both practitioners and patients. The HA used in our patient was very heavy in its molecular characteristics, and it should not be injected into the nasolabial fold. Our patient had a favorable outcome since she received appropriate treatment in less than 72 hours. Prompt recognition and treatment with hyaluronidase are critical to mitigate damage in these cases.
